# Supplementary material for: A zwitterionic near-infrared fluorophore for real-time ureter identification during laparoscopic abdominopelvic surgery
Source: Nat Commun. 2019 Jul 16;10:3118. doi: 10.1038/s41467-019-11014-1 (PMC6635391; doi:10.1038/s41467-019-11014-1)

Supplementary Materials of the Manuscript:

A Zwitterionic Near-Infrared Fluorophore for Real-Time Ureter Identification during Laparoscopic Abdominopelvic Surgery

de Valk et al.

Supplementary Table 1. Demographics healthy volunteers and patients per treatment group.

Healthy volunteers (n=16)

	**0.5 mg ZW800-1 Active drug (n=4) and Placebo (n=2)**	**2.5 mg ZW800-1 Active drug (n=4) and Placebo (n=1)**	**5.0 mg ZW800-1 Active drug (n=4) and Placebo (n=1)**
Sex			
Female (n=8)	4	3	1
Male (n=8)	2	2	4
Age (years)			
Mean	28.8	29.6	28
Median	24	21	24
Range	22–56	18–57	20–48
Height (cm)			
Mean	173.2	172.9	177.5
Median	172.4	169.7	178.7
Range	164.0–185.8	157.0–187.7	162.4–187.1
Weight (kg)			
Mean	66.1	67.18	79.0
Median	66.4	58.35	82.20
Range	59.30–77.10	52.80–97.15	60.75–104.90

Patients (n=12)

	**1.0 mg ZW800-1**	**2.5 mg ZW800-1**	**5.0 mg ZW800-1**
Sex			
Female (n=3)	3	0	0
Male (n=9)	1	4	4
Age (years)			
Mean	54.5	53.5	68.3
Median	56	57.5	71
Range	34–72	30–69	58–73
Height (cm)			
Mean	175.5	182.5	179.0
Median	175.0	179.0	175.5
Range	165.0–187.0	176.5–192.0	174.0–191.0
Weight (kg)			
Mean	85.0	87.2	87.6
Median	82.0	86.15	85.0
Range	82.0–96.0	83.8–92.7	80.0–105.0
Surgical procedure			
	➢ Laparoscopic sigmoid resection<br>➢ Laparoscopic pelvic lymph node dissection<br>➢ Laparoscopic subtotal colectomy<br>➢ Laparoscopic hysterectomy	➢ Laparoscopic sigmoid resection<br>➢ Laparoscopic pelvic lymph node dissection<br>➢ Laparoscopic subtotal colectomy<br>➢ Laparoscopic abdominoperineal resection	➢ Laparoscopic sigmoid resection<br>➢ Laparoscopic pelvic lymph node dissection<br>➢ Laparoscopic low anterior resection (n=2)

Supplementary Table 2. Overview of adverse events.

Dose cohort	System organ class / preferred term	Symptom	Severity	Serious adverse event	Relationship to ZW800-1	Occurrence
**Healthy volunteer study (n=16)**						
0.5 mg ZW800-1						
Patient 1	Skin and subcutaneous tissue disorders (preferred term: skin irritation)	Skin irritation ECG sticker site	Mild	No	Unlikely	Single occasion
Patient 2 (placebo)	Infections and infestations (preferred term: influenza)	Common cold	Mild	No	Unrelated	Single occasion
5.0 mg ZW800-1						
Patient 3	Injury, poisoning and procedural complications (preferred term: procedural complication)	Subcutaneous infusion (administration site)	Mild	No	Unrelated	Single occasion
Patient 4	Nervous system disorders (preferred term: headache)	Headache	Mild	No	Possible	Single occasion
Patient 5	Psychiatric disorders (preferred term: dyssomnia)	Tired	Mild	No	Possible	Single occasion
	Infections and infestations (preferred term: urinary tract infection)	Urinary tract infection	Mild	No	Possible	Single occasion
2.5 mg ZW800-1						
Patient 6	Metabolism and nutrition disorders (preferred term: increased appetite)	Increased appetite	Mild	No	Possible	Single occasion
	Injury, poisoning and procedural complications (preferred term: infusion site discomfort)	Painful infusion site (non-administration arm)	Mild	No	Unlikely	Single occasion
	Injury, poisoning and procedural complications (preferred term: procedural complication)	Subcutaneous infusion (administration site)	Mild	No	Unrelated	Single occasion
Patient 7	Injury, poisoning and procedural complications (preferred term: application site hematoma)	Hematoma cannula site	Mild	No	Unlikely	Single occasion
**Patient study (n=12)**						
5.0 mg ZW800-1						
Patient 1	Injury, poisoning and procedural complications (preferred term: procedural pain)	Dyspnea	Moderate	No	Unrelated	Single occasion
	Injury, poisoning and procedural complications (preferred term: procedural pain)	Chest pain	Mild	No	Unrelated	Single occasion
Patient 2	Injury, poisoning and procedural complications (preferred term: postoperative ileus)	Ileus paralytic	Moderate	No	Unrelated	Single occasion
	Infections and infestations (preferred term: postoperative wound infection)	Wound infection	Mild	No	Unrelated	Single occasion
Patient 3	Gastrointestinal disorders (preferred term: nausea)	Nausea	Moderate	No	Unrelated	Single occasion

Supplementary Figure 1. Pharmacokinetics (blood) healthy volunteers and patients combined per dose group. Note: One Phase 2 surgical patient in the 2.5 mg dose (graph B) had a slightly decreased kidney function resulting in the deviation observed in the curve. All other subjects had normal kidney function (eGFR >90).

A)

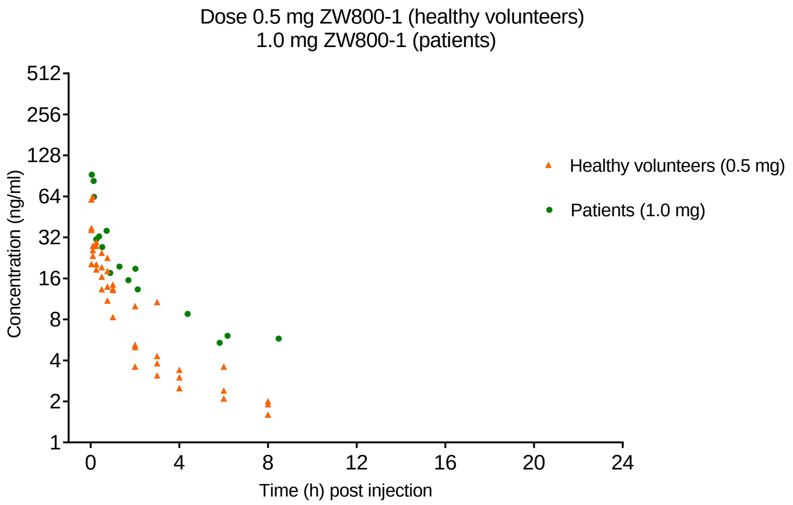

B)

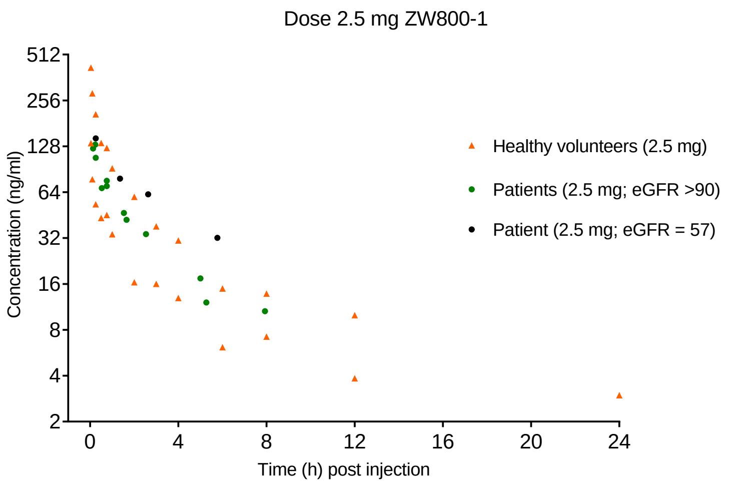

C)

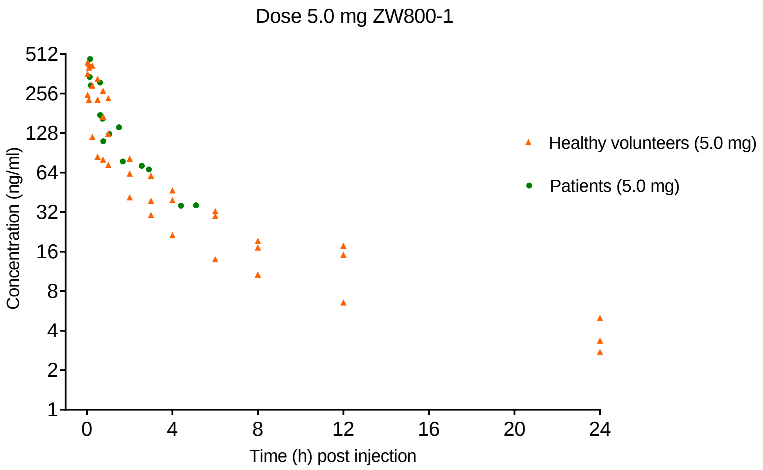

Supplementary Figure 2. Fluorescence of the skin in healthy volunteers. Figure A depicts a graph that illustrates the SBR measured over time in the three dosing cohorts, where the baseline measurement of skin was used as 'background' in the SBR calculations. The values represent the mean ± SD (n= 3 healthy volunteers each group). Figure B shows an example of skin perfusion perceived in a healthy volunteer who received 2.5 mg ZW800-1. Imaging of the skin was performed with the FLARE® imaging system.

A)

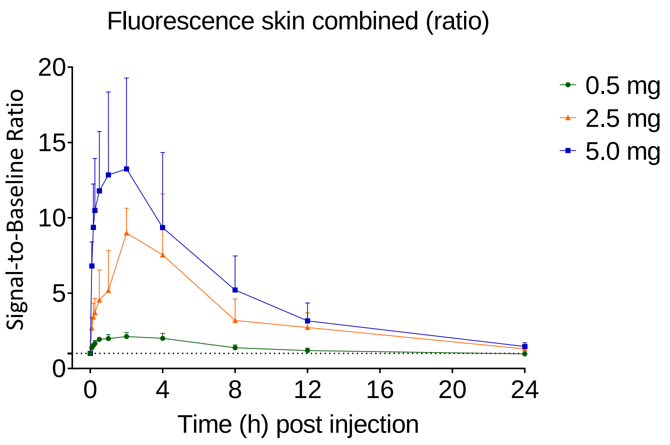

B)

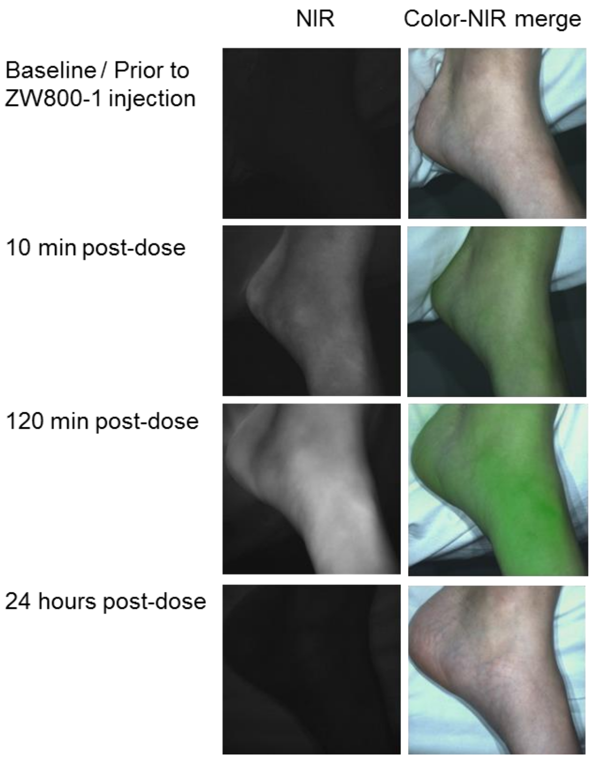

Supplementary Figure 3. Cumulative urine excretion ZW800-1 healthy volunteers. The values represent the mean ± SD (n=4 healthy volunteers in 0.5 mg and 3 healthy volunteers in 2.5 mg and 5.0 mg, respectively).

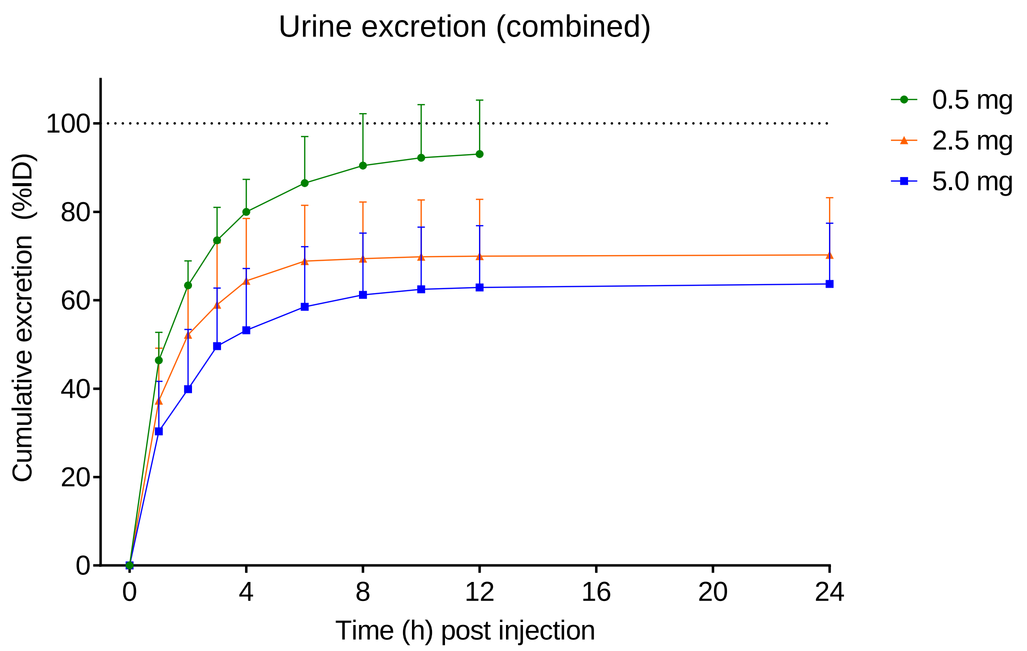

Supplement: Supplementary file 1 — Supplementary Information [file 41467_2019_11014_MOESM1_ESM.pdf]
