# Supplementary material for: A zwitterionic near-infrared fluorophore for real-time ureter identification during laparoscopic abdominopelvic surgery
Source: Nat Commun. 2019 Jul 16;10:3118. doi: 10.1038/s41467-019-11014-1 (PMC6635391; doi:10.1038/s41467-019-11014-1)
Supplement: Supplementary file 3 — Description of Additional Supplementary Files [file 41467_2019_11014_MOESM3_ESM.docx]

**Description of Supplementary Files**

**File Name:** Supplementary Movie 1

**Description:** Vascular flush occurring 8-10 seconds after intravenous bolus injection of 0.5 mg ZW800-1.

**File Name:** Supplementary Movie 2

**Description:** Near-infrared fluorescence imaging of the ureter with Da Vinci® Firefly. This movie presents a case where the surgeon thought he had identified the ureter. However, the structure initially identified was not actually the ureter, as confirmed by ZW800-1 injection. During inspection of the initial structure, fluorescence subtly emerged under the structure, identifying the ureter.

**File Name:** Supplementary Movie 3

**Description:** Near-infrared fluorescence imaging of the ureters in a patient undergoing laparoscopic abdominal surgery with 2.5 mg ZW800-1. Surgery was performed with the Olympus® NIR imaging system.
