# Supplementary material for: A zwitterionic near-infrared fluorophore for real-time ureter identification during laparoscopic abdominopelvic surgery
Source: Nat Commun. 2019 Jul 16;10:3118. doi: 10.1038/s41467-019-11014-1 (PMC6635391; doi:10.1038/s41467-019-11014-1)
Supplement: Supplementary file 7 — Reporting Summary [file 41467_2019_11014_MOESM7_ESM.pdf]

nature research

Corresponding author(s): K.S. de Valk
A.L. Vahrmeijer

Last updated by author(s): Apr 16, 2019

Reporting Summary

Nature Research wishes to improve the reproducibility of the work that we publish. This form provides structure for consistency and transparency in reporting. For further information on Nature Research policies, see Authors & Referees and the Editorial Policy Checklist.

Statistics

For all statistical analyses, confirm that the following items are present in the figure legend, table legend, main text, or Methods section.

n/a	Confirmed	
☐	☒	The exact sample size (*n*) for each experimental group/condition, given as a discrete number and unit of measurement
☒	☐	A statement on whether measurements were taken from distinct samples or whether the same sample was measured repeatedly
☐	☒	The statistical test(s) used AND whether they are one- or two-sided<br>*Only common tests should be described solely by name; describe more complex techniques in the Methods section.*
☒	☐	A description of all covariates tested
☒	☐	A description of any assumptions or corrections, such as tests of normality and adjustment for multiple comparisons
☒	☐	A full description of the statistical parameters including central tendency (e.g. means) or other basic estimates (e.g. regression coefficient) AND variation (e.g. standard deviation) or associated estimates of uncertainty (e.g. confidence intervals)
☐	☒	For null hypothesis testing, the test statistic (e.g. *F*, *t*, *r*) with confidence intervals, effect sizes, degrees of freedom and *P* value noted<br>*Give P values as exact values whenever suitable.*
☒	☐	For Bayesian analysis, information on the choice of priors and Markov chain Monte Carlo settings
☒	☐	For hierarchical and complex designs, identification of the appropriate level for tests and full reporting of outcomes
☒	☐	Estimates of effect sizes (e.g. Cohen's *d*, Pearson's *r*), indicating how they were calculated

Our web collection on statistics for biologists contains articles on many of the points above.

Software and code

Policy information about availability of computer code

Data collection	Data was collected and entered in a designed Promasys database.
Data analysis	PK data was analyzed with PK variable programming dedicated for PK analysis (R 2.12.0 for Windows). Imaging data was analyzed using ImageJ 1.518 (National Institute of Health).

For manuscripts utilizing custom algorithms or software that are central to the research but not yet described in published literature, software must be made available to editors/reviewers. We strongly encourage code deposition in a community repository (e.g. GitHub). See the Nature Research guidelines for submitting code & software for further information.

Data

Policy information about availability of data

All manuscripts must include a data availability statement. This statement should provide the following information, where applicable:

- Accession codes, unique identifiers, or web links for publicly available datasets
- A list of figures that have associated raw data
- A description of any restrictions on data availability

All study data are presented in the manuscript and supplementary materials. Additional raw data that support the findings of this study are available from the corresponding author upon reasonable request.

Field-specific reporting

Please select the one below that is the best fit for your research. If you are not sure, read the appropriate sections before making your selection.

☒ Life sciences ☐ Behavioural & social sciences ☐ Ecological, evolutionary & environmental sciences

For a reference copy of the document with all sections, see nature.com/documents/nr-reporting-summary-flat.pdf

Life sciences study design

All studies must disclose on these points even when the disclosure is negative.

Sample size	The Phase 1 study was designed using commonly accepted subjects per group. For the Phase 2 study, the sample size was not based on statistical power considerations due to the exploratory nature of the study.
Data exclusions	In the Phase 1 study, 2 subjects were completely excluded from the PK analysis due to subcutaneous infusion of the study drug. Blood PK data from one subject was excluded because blood was erroneously drawn from the ZW800-1 infusion cannula, tampering with the accuracy of the data. In the Phase 2 study, all the PK blood results were included in the analysis.
Replication	The pharmacokinetic analysis in both Phase 1 and Phase 2 are the same, only a different population (patients instead of healthy volunteers) and setting (surgery instead of controlled environment) were used.
Randomization	The Phase 1 study was randomized. Subjects were randomized in a consecutive order. The randomization code was generated using SAS 9.4 for Windows by a study independent statistician. The Phase 2 study was not randomized.
Blinding	The Phase 1 study was blinded. The study was performed double-blinded. The investigator, study staff, subjects were blinded to the treatment until end of the study. The investigational drug and matching placebo were packaged indistinguishably. Syringes were wrapped in foil and the iv cannulas were covered during dosing and flushed directly after injection with saline. The same independent physician administered the drug or placebo and performed the fluorescence imaging of the skin. The phase 2 study was not blinded.

Reporting for specific materials, systems and methods

We require information from authors about some types of materials, experimental systems and methods used in many studies. Here, indicate whether each material, system or method listed is relevant to your study. If you are not sure if a list item applies to your research, read the appropriate section before selecting a response.

Materials & experimental systems

n/a	Involved in the study	
☒	☐	Antibodies
☒	☐	Eukaryotic cell lines
☒	☐	Palaeontology
☒	☐	Animals and other organisms
☐	☒	Human research participants
☐	☒	Clinical data

Methods

n/a	Involved in the study	
☒	☐	ChIP-seq
☒	☐	Flow cytometry
☒	☐	MRI-based neuroimaging

Human research participants

Policy information about studies involving human research participants

Population characteristics	In the Phase 1 study, healthy volunteers were included and were healthy based on the medical screening. In the Phase 2 study patients scheduled for a laparoscopic surgery of the lower abdomen were included.
Recruitment	The healthy volunteers in the phase 1 study were recruited through a volunteer research database. The patients in the phase 2 study were recruited through multidisciplinary patient meetings or through the surgeon.
Ethics oversight	Both the healthy volunteer and patient studies were approved by a certified medical ethics review board and complied with all relevant ethical regulations. The medical ethics review board Stichting BEBO in Assen, the Netherlands, approved the healthy volunteer study and the medical ethics review board METC LUMC in Leiden, the Netherlands approved the patient study.

Note that full information on the approval of the study protocol must also be provided in the manuscript.

Clinical data

Policy information about clinical studies

All manuscripts should comply with the ICMJE guidelines for publication of clinical research and a completed CONSORT checklist must be included with all submissions.

Clinical trial registration	The healthy volunteer and patient studies are registered in the European Clinical Trials Database under numbers 2016-003919-35 and 2017-001954-32, respectively, as well as in the Netherlands Trial Register under ID NL7209.
Study protocol	Study protocol is not accessible online, as the study protocol has never been published. The full study protocol is available at the study site.
Data collection	Data was collected and entered in a designed Promasys database. The healthy volunteers in the phase 1 study were recruited through a volunteer research database and the patients in the phase 2 study were recruited through multidisciplinary patient meetings or through the surgeon. The volunteers/patients were given as much time as needed to think about study participation, however they usually decided within 1-2 days after receiving the patient information sheet of the study. After informed consent, data was collected for the study.
Outcomes	Phase I study:<br>Primary objective was to determine safety and tolerability of a single dose of ZW800-1 in healthy volunteers. Secondary objective was to determine the pharmacokinetics of a single dose of ZW800-1. The outcome of safety and tolerability was assessed by the occurrence of adverse events, changes in clinical laboratory parameters, vital signs, ECG and physical examinations. The pharmacokinetics was determined by measuring fluorescence in the blood and urine samples.<br><br>Phase II study:<br>Primary objective was to assess the feasibility of ZW800-1 in intraoperative detection of the urinary tract using the NIR fluorescence imaging system. Secondary objective was to define the optimal dose of ZW800-1 for intraoperative imaging of the urinary tract. The outcome of these objectives were determined by performing a dose escalation and measuring the signal-to-background ratio to find the optimal dose for imaging.
